# Assessing the impact of the iPeer2Peer program for adolescents with juvenile idiopathic arthritis: a mixed-methods randomized controlled trial

**DOI:** 10.1186/s12969-024-01052-5

**Published:** 2024-12-27

**Authors:** Fareha Nishat, Lauren Kelenc, Roberta Berard, Ciaran Duffy, Brian Feldman, Paula Forgeron, Adam M. Huber, Nadia Luca, Heinrike Schmeling, Lynn Spiegel, Lori Tucker, Karen Watanabe-Duffy, Tieghan Killackey, Chitra Lalloo, Brittany Wiles, Anya Nair, Sofia Olaizola, Brenna McDermott, Farideh Tavangar, Sara Ahola Kohut, Jennifer N. Stinson

**Affiliations:** 1 Child Health Evaluative Sciences, Research Institute, The Hospital for Sick Children, 686 Bay Street, Room 06.9715, Toronto, ON M5G 0A4 Canada; 2 Division of Rheumatology, Children’s Hospital, London Health Sciences Centre, London, ON Canada; 3 Children‘s Hospital of Eastern Ontario Research Institute, Ottawa, ON Canada; 4 Department of Rheumatology, The Hospital for Sick Children, Toronto, ON Canada; 5 School of Nursing, Faculty of Health Sciences, University of Ottawa, Ottawa, ON Canada; 6 Division of Pediatric Rheumatology, IWK Health Centre, Halifax, NS Canada; 7 Division of Rheumatology, Children‘s Hospital of Eastern Ontario, Ottawa, ON Canada; 8 Alberta Children’s Hospital, Calgary, AB Canada; 9 Section of Rheumatology, Department of Pediatrics, Cumming School of Medicine, University of Calgary, Calgary, AB Canada; 10 Division of Rheumatology, British Columbia Children’s Hospital, Vancouver, BC Canada; 11 University Health Network, Toronto, Canada; 12 Lawrence S. Bloomberg Faculty of Nursing, University of Toronto, Toronto, Canada; 13 Division of Gastroenterology Hepatology and Nutrition, The Hospital for Sick Children, Toronto, ON Canada

**Keywords:** Online, Peer support, Randomized control trial, Adolescent, Self-management, Juvenile idiopathic arthritis

## Abstract

**Background:**

Juvenile Idiopathic Arthritis (JIA) is a chronic pediatric illness, whereby youth experience physical, emotional and psychosocial challenges that result in reduced health related quality of life (HRQL). Peer mentoring has been shown to improve disease self-management in adults with chronic conditions, with mixed results in younger populations. Building on our pilot work – which supported the feasibility and initial effectiveness of the iPeer2Peer program – the objective of this study was to assess the clinical effectiveness of the program in youth with JIA through a waitlist randomized controlled trial.

**Methods:**

Eighty-one youth (aged 12–18) were randomized to the intervention group and matched with trained peer mentors (18–25 years; successfully managing their JIA), completing of up to ten 30-min video calls over a 15-week period. Eighty-three youth in the control group received standard care. Outcome assessments occurred at enrollment, 15 weeks post randomization and 6-months post randomization. The primary outcome was self-management, measured using the TRANSITION-Q. Secondary outcomes were HRQL, pain, emotional distress, disease knowledge, self-efficacy, and perceived social support. These were assessed using linear mixed effects models. Content analysis of semi-structured interviews and focus groups was used to assess satisfaction with the program with mentors and mentees upon study completion.

**Results:**

In total, 164 youth (mean age 14.4 ± 1.9 years, 78% female) were randomized to the study. The proposed sample size was not reached due to challenges in recruitment, likely impacted by the COVID-19 pandemic. The iPeer2Peer program did not show significant improvement in self-management (*p* = 0.7), or any of the secondary outcomes. Three key categories emerged from content analysis: (1) Fulfillment and Support Through Shared Experience, (2) Enhancing Program Delivery and (3) Strategies to Boost Engagement. These findings highlight that mentees valued the ability to converse with mentors who empathized with their disease experience, while mentors found it fulfilling to support mentees, and noted that they could have benefited from this type of support themselves.

**Conclusion:**

While the iPeer2Peer did not result insignificant changes in clinical outcomes, both mentors and mentees were satisfied with the program and felt that mentorship provided real-world benefits for disease management and overall wellbeing.

**Trial registration:**

ClinicalTrials.gov, NCT03116763. Registered 31, March 2017, https://www.clinicaltrials.gov/study/NCT03116763

## Background

Juvenile Idiopathic Arthritis (JIA) is associated with an array of physical (e.g., pain, stiffness, and fatigue), emotional (e.g., anxiety, and depression) and social (e.g., decrease activity engagement, peer relationship changes) challenges that impact various aspects of an adolescent’s health-related quality of life (HRQL) [1–6]. These challenges often disrupt sleep, reduce overall activity levels, and interfere with young adults’ academic and occupational endeavors, leading to absenteeism and diminished productivity [1–7]. For adolescents with JIA, these challenges can lead to fewer interactions with their peers, hindering friendships and creating a sense of social isolation, thus impacting their development, autonomy and disease management skills [3, 4, 6]. The developmental period of adolescence is further complicated by the impending transition into adult-oriented healthcare systems [8]. Adolescents with JIA are expected to assume greater responsibility for managing their disease, which can be complex and may require multiple concurrent treatments over extended periods [8]. This increased responsibility can be daunting, underscoring the importance of social support in facilitating this transition. While there are existing web-based and app-based self-management interventions for adolescents with JIA, these are rarely delivered through peers [9].

Peer support, which involves emotional, appraisal, and informational help from peers who share similar health experiences, has been associated with improved health outcomes in adults with chronic illness [10–12]. This establishes peer mentoring as a promising strategy to bolster self-management and reduce feelings of isolation in adult populations and warrants exploration among younger populations. For adolescents with JIA, many have never met another person with the same condition, and they often lack access to adolescent-directed education and peer support. These experiences are compounded by additional barriers such as limited availability of services, especially in rural areas, language barriers, long wait times, and the associated costs of attending specialized clinics [7, 13–17]. While the quantitative evidence for peer support in young people with chronic conditions is mixed, young people consistently highlight the utility such programs qualitatively [18–20].

iPeer2Peer is an innovative solution to the need for peer support that leverages online communication platforms to deliver peer mentoring [21–24]. This program aims to enhance self-management skills and social engagement among adolescents with JIA, ultimately improving their HRQL [21–24]. Building on the results of our successful pilot randomized control trial (RCT) [21] – which supported the feasibility and initial effectiveness of the program in improving self-management with 30 adolescents with JIA – we aimed to conduct a full-scale RCT to evaluate the impact of the iPeer2Peer program on disease self-management and other clinical outcomes, as well as describe the satisfaction with the program.

## Methods

### Trial design

A two-arm waitlist RCT design with a 1:1 allocation ratio with repeated measures was used to evaluate the effectiveness of the iPeer2Peer program. The trial was registered on ClinicalTrials.gov (NCT03116763) and approved by research ethics boards at each site.

### Participants

Participants were recruited from six tertiary care pediatric centers in four Canadian provinces: The Hospital for Sick Children (SickKids), Children’s Hospital for Eastern Ontario, BC Children’s Hospital, IWK Health Centre, Alberta Children’s Hospital, and London Health Sciences, between August 2017 and June 2022. Adolescents were included in the study if they were: (a) aged 12–18 years old, (b) rheumatologist-diagnosed with JIA according to International League of Associations for Rheumatology (ILAR) criteria, [25] (c) able to speak and read English, (d) able to access a computer, smartphone or tablet capable of using Skype software, and (e) willing and able to complete online measures***.*** They were excluded if they: (a) had significant cognitive impairments and/or (b) had major co-morbid illnesses (medical or psychiatric conditions) likely to influence HRQL assessment, and/or (c) were participating in other peer support or self-management interventions.

Participants were recruited through two methods: (1) clinic-based recruitment and (2) remote recruitment. In the first approach, eligible patients identified through clinic appointments were introduced to the study by a healthcare provider in their circle of care. The contact information of interested adolescents was shared with the local research team and informed consent was obtained. In the second approach, eligible participants identified through clinical appointments were approached in two ways, a study information letter or during their telemedicine appointment. The study information letter was mailed or e-mailed to the potential participant and was followed-up with telephone or email contact by the research staff. If they were interested, the research staff scheduled a consent discussion. The discussion occurred over the phone or through secure video call (Zoom Healthcare [26]) with the patient. For telemedicine appointments that occurred through secure videoconferencing, the patient’s clinician introduced the research team to the interested study candidate. The research staff then discussed the study and began the consent process for interested participants.

### Intervention

#### Experimental group

Adolescents in the intervention group received the iPeer2Peer JIA program, a virtual peer mentorship program, in additional to standard medical care. This program consisted of up to 10 one-on-one sessions of Skype video calls (approximately 30 min each) over a 15-week period. Mentor contact outside scheduled sessions was discouraged. Mentors were matched to adolescents based on similar disease profiles (e.g. type of JIA, age of onset, symptoms) and non-disease characteristics (e.g., similar interests and hobbies, education aspirations), with each mentor supporting a maximum of 3 mentees simultaneously. Detailed information on iPeer2Peer program development has been previously reported [21, 22].

#### Peer mentor selection

Peer mentors were young adults living with JIA who had successfully transitioned to adult care and demonstrated ability to self-manage their condition. Potential mentors were nominated by healthcare teams at each site and were screened for interest and eligibility by study staff. Mentor inclusion criteria were: (1) between the ages of 18 and 26 years, (2) rheumatologist-diagnosed with JIA according to ILAR criteria [25] (3) nominated by a member of their health care team as a potentially good mentor, (4) self-reported adherence to current treatment plan (80–100% compliance), (5) self-reported successful transition to an adult rheumatologist, (6) no active psychological disorder or a stable psychological disorder with follow-up by a physician/psychologist/psychiatrist, (7) self-reported self-efficacy in their ability to manage their JIA related symptoms, (8) willingness to commit to training (20 h) and mentoring participants (once paired with mentee ~ 30 min calls over a period of 15 weeks), and (9) self-reported good communication skills.

#### Peer mentor training

**Fig. 1:**
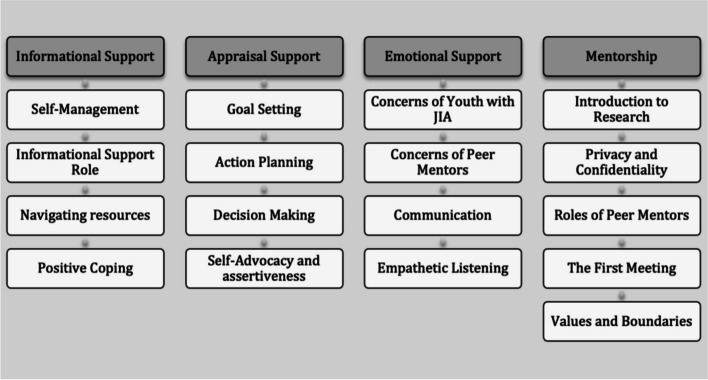
Mentor training outline

All mentors completed a 2-day training course (Fig. 1) led by research team members from SickKids prior to beginning the program. Training comprised of lectures, active group discussion, case examples, small group activities and role-play activities. All peer mentors received a guidebook including all training materials, additional resources and reading lists.

### Control group

The control group received standard care (basic disease education, some transition guidance) as usually provided at each site without the iPeer2Peer program [27]. Following completion of all endpoint measures, control participants were offered the intervention.

### Outcomes

Outcome data were collected via self-reported questionnaires from the adolescent, measured at three time points, baseline (T1; after consent; before randomization), after program completion (T2; 15-weeks after randomization) and 6 months (T3; 6 months post randomization). All questionnaires were completed online through the secure web-based system REDCap [28] hosted at the SickKids.

#### Quantitative outcomes

The a priori primary outcome focused on assessing the effectiveness of the iPeer2Peer program as evaluated using the TRANSITION-Q [29]. The TRANSITION-Q is a psychometrically sound 14-item measure of self-management skills that is reliable and valid (described in Table 1) [29]. Secondary effectiveness outcomes included: HRQL, pain, emotional distress, disease knowledge, self-efficacy, and perceived social support, see Table 1 for descriptions of each tool. Adolescents received gift cards for completion of outcome measures at each time point ($10 at T1, $15 at T2 and $20 at T3).

**Table 1:** Primary and Secondary Effectiveness Measures

Outcome	Measurement Tool	Description
Self-management	TRANSITION-Q	A 14-item generic tool to capture self-management skills in adolescents (12–18 year olds) with chronic conditions. Response options are: 2 (“*Always”*), 1 (“*Sometimes”*) or 0 (“*Never”*). Items are transformed to a 0–100 scale, with higher scores indicate greater self-management. Psychometrics: Person Separation Index = 0.82; no differential item function by age or gender; low residual correlations between items; Cronbach's α = 0.85; test–retest reliability = 0.90. This tool has also been used in JIA and pediatric pain populations [48, 49].
HRQL	PedsQL Arthritis Module [50]	A 22-item multidimensional scale (pain and hurt, daily activities, treatment, worry, and communication), rated using a 5-point Likert scale ranging from 0 (“*never a problem”*) to 4 (“*almost always a problems”*). Items are reverse scored and linearly transformed to a 0–100 scale, with higher scores indicating better HRQOL. Psychometrics: internal consistency α = 0.75–0.86, ability to distinguish between healthy children and children with arthritic conditions, and responsiveness through patient change over time
Pain	PROMIS Pediatric Pain Interference – Short Form [51, 52]	A 8-item scale used to measure self-reported consequences of pain among pediatric populations with chronic conditions, rated using a 5-point Likert scale ranging from 1 (“*never”*) to 5 (“*almost always”*). Total raw scores are transformed to a standardized T-Score (population mean = 10, standard deviation = 10), with higher scores suggesting greater pain interference. Psychometrics: Item Response Theory was used to develop this tool, it has the ability to distinguish between inactive and active disease in adolescents with JIA, high pairwise correlations between patient and parent dyads (ICC = 0.8) [51, 52]. This tool has also been used in JIA and pediatric pain populations [52, 53].
Emotional distress	Screen for Child Anxiety Related Disorders [54]	A 41-item scale used to screen for anxiety disorders, rated on a 3-point scale: 0 (*Not True/Hardly True*), 1 (*Somewhat True or Sometimes True*), 3 (*Very True or Often True*). Includes 5 subscales: Pain Disorder or Significant Somatic Symptoms, Generalized Anxiety Disorder, Separation Anxiety Disorder, Social Anxiety Disorder and School Avoidance. Summed scores can range from 0–82, higher scores indicate higher levels of anxiety symptoms, scores ≥ 25 may indicate the presence of an anxiety disorder. Psychometrics: Good internal consistency (α = 0.95) and discriminant validity are reported. This tool has also been used in JIA and pediatric pain populations [55, 56].
	Center for Epidemiologic Studies Depression Scale [57]	A 20-item scale that assess depressive symptoms in individuals between 6–17 years of age, rated on a 4-point scale, ranging from 0 (*Not At All*) to 3 (*A Lot*) – 4 items are reverse scored. Summed scores can range from 0 to 60, with higher scores suggesting increasing levels of depression. Psychometrics: internal consistency ranging from α = 0.84–0.92, concurrent validity with other validated depressive measurement tolls and good test–retest reliability [58–60]. This tool has also been used in JIA and pediatric pain populations [61].
Disease knowledge	Medical Issues, Exercise, Pain and Social Support [MEPS] Questionnaire [62]	A 23-item scale (4 subscales: knowledge, pain, social, exercise) used to assess JIA-specific disease knowledge, items are rated on a 11 point number rating scale [1–10]. Mean total scores range from 0 – 230, mean subscale scores range from 0 – 90 depending on the number of questions in each subscale, with higher scores indicating greater knowledge. Psychometrics: evidence of construct validity as well as test–retest reliability [62, 63].
Self-efficacy	Children’s Arthritis Self-Efficacy [64]	A 11-item scale (3 subscales: activity, symptom and emotions) that assesses an adolescents perceived ability to control or manage salient aspects of living with JIA. Items are rated on a 5-point scale, ranging from 1 (“*Not at all sure*”) to 5 (“*Very sure*”). Mean scores range from 11 – 55, mean subscale score range from 1–5, with higher scores indicating better self-efficacy [64, 65]. Psychometrics: evidence of construct validity and all 3 subscale show reasonable reliability (α = 0.85–0.90) [64, 65].
Perceived social support	PROMIS Pediatric Peer Relationship Scale – Short Form [66]	A 8-item scale used to measure the quality of self-reported peer relationships. Items are rated using a 5-point Likert scale ranging from 1 (“*never”*) to 5 (“*almost always”*). Total raw scores are transformed to a standardized T-Score (population mean = 10, standard deviation = 10), with higher scores suggesting better peer relationships. Psychometrics: Item Response Theory was used to develop this tool, good test–retest reliability (ICC = 0.81), internal consistency (α = 0.83–0.95), good construct validity and responsiveness [66, 67]. This tool has also been used in JIA and pediatric pain populations [52, 53].

#### Qualitative outcome

Satisfaction with the program was assessed qualitatively through (1) semi-structured interviews with a random subset (approximately 15% of the sample) of adolescents in the intervention group at the end of the study and (2) through semi-structured focus group with mentors who agreed upon study completion.

### Sample size

Sample size calculation was determined based on the primary outcome of self-management as measured by TRANSITION-Q, to detect a small effect size (Cohen’s d = 0.25), as well as published means and standard deviations from a population of adolescent (12 to 18 years of age) with chronic conditions. Assuming a two-tailed type I error of 0.05, a sample size of 222 people (111 in each group) was used to achieve 80% power to detect a difference of 3.3 points on the 0-to-100-point scale. To account for a potential 15% dropout rate, the total sample size was increased to 262 (131 per group; i.e., 111/0.85).

### Randomization

A secure, web-based randomization service (www.randomize.net) was used to allocate participants to trial groups and ensure hidden allocation. Following consent and baseline measurement, the study coordinator entered the participant study number and stratification variables (i.e., disease activity [30] and study center) to the randomization service for group allocation.

### Blinding

The randomization process was blinded to the principal investigator and co-investigators to reduce the chance of experimenter bias. Participants and mentors were asked not to discuss their study involvement with others until study completion.

### Quantitative analysis

Data were analyzed using STATA version 15.1 and R version 4.2.0 [31, 32]. For mentees, demographic and disease characteristics were described with mean and standard deviation for continuous variables and raw counts and percentages for categorical variables by allocation group. As per an intent-to-treat approach, all participants were included in the final analysis according to the allocation group to which they were randomized. Linear mixed models using maximum likelihood estimation were used to assess the effects of the iPeer2Peer program on primary and secondary effectiveness outcomes, with a binary indicator for allocation group, 3-level indicator for assessment time points and binary indicator for sex as fixed effects and intercepts for each participant as random effects, with a significance level of 0.05 for the primary outcome. A Bonferroni-adjusted alpha level of 0.007 was used to maintain an overall level of 0.05 for all secondary outcomes.

#### Qualitative analysis

Individual interviews and focus groups were audio recorded, transcribed and analyzed using directed content analysis (a process by which a coding schema is designed using theory or relevant research findings as opposed to inductively). [33, 34] Three team members (BM, SO, AN) developed an initial coding framework based on reviews of all transcripts and informed by coding structures of other iPeer2Peer studies. This framework included codes that represented meaningful units (phrases to several sentences) of text, which was iterated upon during the analysis of all transcripts. Three members (BM, SO, ET) coded all transcripts independently, such that each transcript was coded twice. Team members communicated with each other if there was a need to develop new codes to capture relevant content. After completion of coding, three team members (BM, SO, FN) organized the codes into overarching categories, with supervision from senior team members (TK, JS, SAK). All analyses were completed on Dedoose, a cross-platform cloud-based application for analyzing qualitative data [35].

## Results

### Participants

**Fig. 2:**
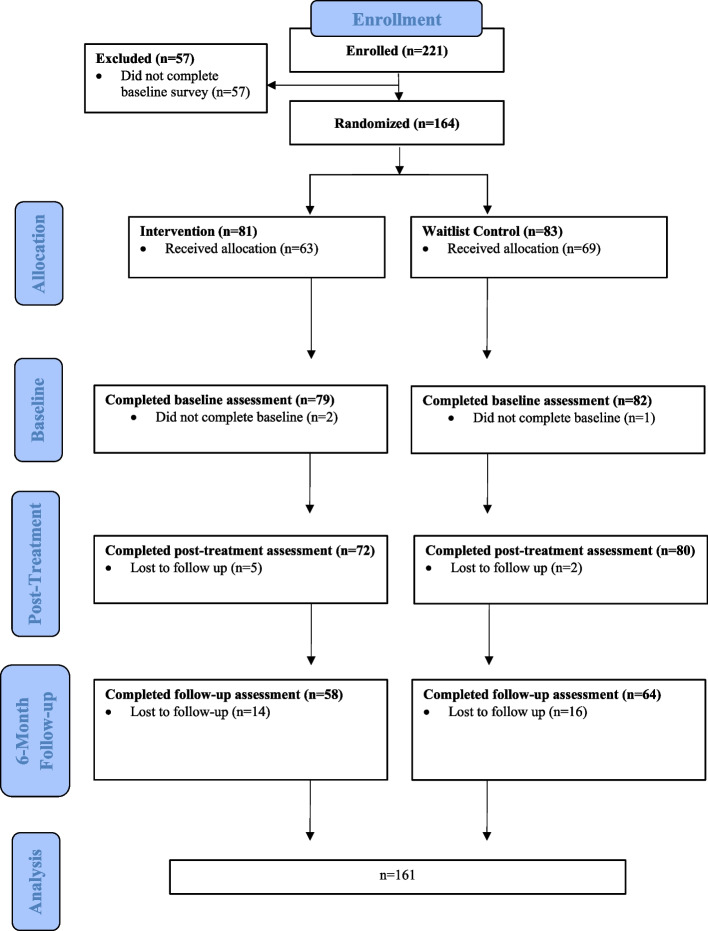
Consolidated standards of reporting trials flow diagram. Note. Screening and eligibility data collected during recruitment was not available from study sites. As a result, it is not provided in the flow diagram

Participant enrollment began in August 2017 and ended June 2022, with final follow up completed in May 2023. The COVID-19 pandemic resulted in a longer than expected recruitment period. The Consolidated Standards of Reporting Trials (CONSORT) diagram is presented in Fig. 2. Screening and eligibility data collected during recruitment was not available from all study sites, as such it is not provided in the flow diagram. The required sample size was not met, 164 participants were randomized, of which 81 received the iP2P program and 83 received the waitlist control. The analyzed sample includes 161 participants, as two participants were randomized despite not completing baseline questionnaires. The recruitment rate was 74% (164/221). The retention rate at T2 for the intervention group was 89% (72/81) and 96% (80/83) for the control group, at T3 it was 72% (58/81) for the intervention group and 77% (64/83) for the control. Most participants were enrolled and randomized before the COVID-19 pandemic (Total 112/161; Intervention: 56/79; Control 56/82). No adverse events were reported by participants over the duration of the project.

Participant demographic and baseline characteristics are presented in Table 2. Most mentees were female (125/161, 77%), aged 12–18 years, with an average age of 14.2 years (Standard Deviation [SD] 1.9). Most mentees had either Polyarthritis (47/161) or Oligoarthritis (45/161), with an average disease duration of 5.95 years (SD 4.6).

**Table 2:** Adolescent demographics and disease characteristics

Characteristic	Intervention (*n* = 79)	Control (*n* = 82)	Total (*n* = 161)
Age in years, mean (sd); range	14.5 (1.96); 12–18	14.3 (1.84); 12–18	14.4 (1.89); 12–18
Gender, n(%)			
* Female*	62 (79)	63 (77)	125 (78)
* Male*	17 (21)	19 (23)	36 (22)
Duration of illness in years, mean(sd)	5.7 (5.10)	6.2 (4.59)	5.9 (4.6)
JIA Type, n(%)			
* Oligoarthritis*	23 (29)	22 (27)	45 (28)
* Polyarthritis*	20 (25)	27 (33)	47 (29)
* Systemic Arthritis*	9 (11)	9 (11)	18 (11)
* Enthesitis-related Arthritis*	10 (13)	14 (17)	24 (15)
* Psoriatic Arthritis*	10 (13)	5 (6)	15 (9)
* Undifferentiated or Unclassified*	4 (5)	2 (2)	6 (4)
* Missing*	3 (4)	3 (4)	6 (4)

### Adherence to the iP2P program

Of those who completed at least one call (*n* = 53/79), the average number of calls completed was 6. Both male [4.05 (SD 4.33)] and female [4.24 (SD 3.88)] participants completed on average the same number of calls. Among the control group, 10 mentees used the iPeer2Peer program after the 15-week study period ended.

### Quantitative outcomes – clinical effectiveness

Table 3 summarizes the medians (25th percentile, 75th percentile) of the clinical effectiveness outcomes by allocation group, no statistical differences were identified between groups at baseline. The Mann–Whitney U test was used due to the non-normal distribution of the outcomes.

**Table 3:** Adolescent reported primary and secondary outcomes, median distribution at baseline, 15 weeks and 6 months post randomization n=161

	**Group** **median (25th percentile, 75th percentile)**		
**Outcome**	**Control** ***N*** ** = 82**	**Intervention** ***N*** ** = 79**	***P*** **-value** ^**1**^
**Transition-Q** (range: 0 – 100. higher scores indicate better self-management skills)			
Baseline	56.0 (50.0, 63.0)	61.0 (50.0, 68.0)	0.441
15 weeks	58.0 (47.8, 68.0)	58.0 (50.0, 70.0)	0.613
6 months	58.0 (50.0, 66.5)	61.0 (50.0, 73.0)	0.656
**Pain interference** (range: 34–78, higher scores indicate higher pain intensity)			
Baseline	52.7 (40.6, 57.6)	53.7 (45.8, 59.5)	0.416
15 weeks	54.2 (40.6, 59.5)	51.7 (41.7, 57.6)	0.514
6 months	48.4 (39.2, 58.5)	53.7 (43.4, 59.0)	0.284
**Anxiety** (range: 0–82, higher scores indicate greater anxiety symptoms)			
Baseline	26.0 (12.0, 39.0)	22.0 (12.2, 36.0)	0.401
15 weeks	28.0 (14.3, 37.0)	21.0 (11.0, 40.5)	0.577
6 months	22.0 (9.3, 34.8)	24.0 (11.5, 40.0)	0.430
**Depression** (0 to 60, higher scores indicate greater levels of depression)			
Baseline	14.0 (8.0, 25.0)	13.0 (7.0, 23.0)	0.574
15 weeks	16.0 (11.0, 24.0)	14.0 (8.0, 22.5)	0.279
6 months	14.0 (9.0, 24.0)	16.0 (7.5, 26.0)	0.737
**Subscales of PedsQL**(all subscales range from 0–100, higher scores indicate lower problems)			
**Pain**			
Baseline	56.3 (37.5, 75.0)	50.0 (31.3, 78.1)	0.466
15 weeks	68.8 (50.0, 81.3)	50.0 (40.6, 68.8)	**0.042**
6 months	62.5 (50.0, 81.3)	46.9 (35.9, 75.0)	**0.009**
**Activities**			
Baseline	100.0 (80.0, 100.0)	100.0 (77.5, 100.0)	0.502
15 weeks	100.0 (85.0, 100.0)	95.0 (67.5, 100.0)	0.310
6 months	100.0 (85.0, 100.0)	95.0 (78.8, 100.0)	0.143
**Treatment**			
Baseline	75.0 (57.1, 89.3)	75.0 (58.9, 82.1)	0.800
15 weeks	78.6 (61.6, 89.3)	67.9 (53.6, 87.5)	0.313
6 months	82.1 (64.3, 89.3)	73.2 (57.1, 89.3)	0.144
**Worry**			
Baseline	58.3 (41.7, 83.3)	58.3 (41.7, 75.0)	0.552
15 weeks	66.7 (50.0, 83.3)	58.3 (45.8, 75.0)	0.260
6 months	66.7 (50.0, 91.7)	58.3 (41.7, 75.0)	0.065
**Communication**			
Baseline	66.7 (50.0, 83.3)	75.0 (50.0, 91.7)	0.190
15 weeks	75.0 (50.0, 91.7)	75.0 (50.0, 91.7)	0.703
6 months	66.7 (54.2, 91.7)	66.7 (50.0, 83.3)	0.398
**MEPS and subscales** (total score range: 0 – 230; mean subscale scores range: 0 – 90, higher scores indicate greater knowledge)			
**Total score**			
Baseline	118.0 (93.0, 142.0)	115.0 (97.0, 129.0)	0.519
15 weeks	121.0 (99.0, 137.0)	118.0 (104.5, 131.5)	0.937
6 months	127.5 (107.0, 148.2)	114.0 (103.0, 141.0)	0.210
**Knowledge**			
Baseline	37.0 (23.0, 49.0)	36.0 (24.5, 44.5)	0.877
15 weeks	36.0 (28.0, 45.0)	39.0 (27.0, 47.0)	0.544
6 months	42.0 (26.3, 54.0)	38.0 (29.0, 50.0)	0.772
**Pain**			
Baseline	35.0 (27.0, 44.0)	33.0 (23.0, 41.0)	0.227
15 weeks	35.0 (27.0, 42.0)	34.0 (28.5, 41.0)	0.680
6 months	40.5 (30.0, 48.8)	33.0 (25.0, 41.0)	**0.030**
**Social**			
Baseline	19.0 (14.0, 26.0)	20.0 (15.5, 24.0)	0.993
15 weeks	20.0 (16.0, 24.0)	22.0 (17.5, 29.5)	**0.039**
6 months	19.5 (15.0, 26.8)	21.0 (14.0, 27.0)	0.879
**Exercise**			
Baseline	27.0 (19.0, 36.0)	29.0 (20.0, 33.5)	0.800
15 weeks	28.0 (20.0, 34.0)	24.0 (20.0, 31.0)	0.133
6 months	26.0 (20.0, 35.8)	25.0 (20.0, 31.0)	0.441
**Self-efficacy total score and domains** (Mean total scores range: 11 – 55, mean subscale score range: 1–5, higher scores indicating better self-efficacy)			
**Total score**			
Baseline	34.0 (28.5, 40.5)	35.0 (28.0, 44.0)	0.626
15 weeks	36.5 (31.0, 40.0)	36.0 (30.0, 40.0)	0.847
6 months	38.0 (32.0, 46.0)	35.0 (28.0, 41.0)	**0.041**
**Activity**			
Baseline	3.7 (2.7, 4.7)	4.0 (2.7, 4.3)	0.867
15 weeks	3.7 (3.0, 4.7)	3.3 (3.0, 4.0)	0.248
6 months	4.0 (3.0, 5.0)	3.7 (3.0, 4.0)	0.114
**Emotional**			
Baseline	3.0 (2.4, 4.0)	3.5 (2.3, 4.3)	0.274
15 weeks	3.3 (2.8, 4.2)	3.3 (3.0, 4.0)	0.956
6 months	3.5 (2.9, 4.6)	3.3 (2.5, 4.0)	0.088
**Symptoms**			
Baseline	3.0 (2.5, 3.7)	3.0 (2.3, 3.7)	0.852
15 weeks	3.3 (2.8, 3.8)	3.0 (2.8, 3.7)	0.405
6 months	3.5 (3.0, 4.3)	3.2 (2.5, 3.8)	**0.013**
**Perceived social support (**range: 34–78, higher scores indicate better peer reltionships)			
Baseline	44.3 (39.8, 50.9)	46.7 (39.8, 52.6)	0.314
15 weeks	45.5 (39.8, 56.8)	44.9 (37.7, 56.8)	0.670
6 months	45.5 (39.8, 51.8)	45.5 (38.8, 50.9)	0.506

*MEPS *Medical Issues, Exercise, Pain and Social Support, *PedsQL *Pediatric Quality of Life Inventory

In the linear mixed model, the interaction effect of time and group was not significant in any of the models; therefore, we considered each model with main effect of time and group adjusting for sex. Visual inspection of residual plots did not reveal any obvious deviations from homoscedasticity or normality. Self-management skills (measured by TRANSITION-Q) among adolescents in the intervention group did not show a significant difference compared to those in the control group (see Table 4). However, there was a significant time effect at 6 months (β = 3.22, 95% C.I: 1.14, 5.31; *P* = 0.003), illustrating that regardless of group allocation, self-management improved over time. Overall, the effect of intervention on all secondary outcomes was not significant compared to the control group (see Table 4).

**Table 4:** Adolescent reported primary and secondary outcomes, linear mixed models, n=161

Outcome	Predictor	β (95% C.I)	*P*-value
Transition-Q	T1-T2: 15 weeks^1^	0.55 (−1.21, 2.31)	0.539
	T2-T3: 6 months	3.22 (1.14, 5.31)	**0.003**
	Intervention^2^	0.80 (−3.36, 4.95)	0.707
	Sex^3^	3.73 (−1.27, 8.73)	0.145
PROMIS Pain interference	T1-T2: 15 weeks	−0.30(−1.86, 1.25)	0.704
	T2-T3: 6 months	−0.96(−2.60, 0.68)	0.254
	Intervention	1.10 (−1.93, 4.14)	0.477
	Sex	2.03 (−1.64, 5.69)	0.279
Anxiety	T1-T2: 15 weeks	−0.65(−2.72, 1.42)	0.540
	T2-T3: 6 months	−0.42(−2.61, 1.77)	0.709
	Intervention	−1.15(−5.73, 3.43)	0.623
	Sex	12.54 (7.02, 18.05)	**< 0.001**
Depression	T1-T2: 15 weeks	0.26 (−1.35, 1.88)	0.749
	T2-T3: 6 months	1.14 (−0.56, 2.84)	0.189
	Intervention	−0.66(−3.74, 2.42)	0.676
	Sex	6.19 (2.47, 9.91)	**0.001**
Subscales of PedsQL			
* Pain*	T1-T2: 15 weeks	4.26 (1.41, 7.11)	**0.004**
	T2-T3: 6 months	4.42 (1.42, 7.43)	**0.004**
	Intervention	−6.25(−13.91, 1.41)	0.111
	Sex	−3.18(−12.41, 6.05)	0.499
* Activities*	T1-T2: 15 weeks	−2.16(−4.47, 0.15)	0.069
	T2-T3: 6 months	−0.66(−3.09, 1.78)	0.599
	Intervention	−3.50(−8.69, 1.70)	0.189
	Sex	−3.92(−10.19, 2.34)	0.221
* Treatment*	T1-T2: 15 weeks	1.80 (−0.72, 4.32)	0.163
	T2-T3: 6 months	2.71 (0.05, 5.36)	**0.047**
	Intervention	−2.74(−8.17, 2.70)	0.325
	Sex	−5.60(−12.16, 0.96)	0.096
* Worry*	T1-T2: 15 weeks	2.44 (−0.71, 5.60)	0.130
	T2-T3: 6 months	4.97 (1.65, 8.30)	**0.004**
	Intervention	−4.12(−11.67, 3.43)	0.285
	Sex	−7.03(−16.13, 2.07)	0.131
Communication	T1-T2: 15 weeks	3.61 (0.31, 6.91)	**0.033**
	T2-T3: 6 months	2.67 (−0.80, 6.14)	0.134
	Intervention	1.37 (−5.07, 7.82)	0.676
	Sex	−2.60(−10.38, 5.19)	0.513
MEPS total score and subscales			
* Total score*	T1-T2: 15 weeks	3.52 (−0.92, 7.97)	0.122
	T2-T3:: 6 months	7.18 (2.45, 11.91)	**0.003**
	Intervention	−3.02(−11.23, 5.19)	0.472
	Sex	4.56 (−5.35, 14.48)	0.368
* Knowledge*	T1-T2: 15 weeks	1.96 (−0.38, 4.30)	0.102
	T2-T3: 6 months	5.28 (2.79, 7.77)	**< 0.001**
	Intervention	−0.15(−4.40, 4.09)	0.944
	Sex	4.21 (−0.93, 9.34)	0.110
* Pain*	T1-T2: 15 weeks	1.05 (−0.97, 3.07)	0.311
	T2-T3: 6 months	2.31 (0.16, 4.45)	**0.036**
	Intervention	−2.59(−5.81, 0.63)	0.116
	Sex	0.87 (−3.02, 4.77)	0.661
* Social*	T1-T2: 15 weeks	1.13 (−0.05, 2.31)	0.061
	T2-T3: 6 months	0.06 (−1.19, 1.31)	0.927
	Intervention	0.75 (−1.41, 2.91)	0.498
	Sex	3.14 (0.53, 5.75)	**0.019**
* Exercise*	T1-T2: 15 weeks	−0.61(−1.88, 0.65)	0.345
	T2-T3: 6 months	−0.44(−1.79, 0.91)	0.525
	Intervention	−1.01(−3.39, 1.37)	0.407
	Sex	−3.59(−6.47,−0.72)	**0.015**
Self-efficacy total score and domains			
* Total score*	T1-T2: 15 weeks	0.87 (−0.72, 2.47)	0.285
	T2-T3: 6 months	2.28 (0.57, 3.98)	**0.009**
	Intervention	−0.65(−3.22, 1.91)	0.617
	Sex	−0.81(−3.99, 2.38)	0.620
* Activity*	T1-T2: 15 weeks	−0.03(−0.21, 0.14)	0.703
	T2-T3: 6 months	0.12 (−0.06, 0.31)	0.199
	Intervention	−0.13(−0.42, 0.16)	0.394
	Sex	0.01 (−0.35, 0.37)	0.954
* Emotional*	T1-T2: 15 weeks	0.12 (−0.06, 0.31)	0.195
	T2-T3: 6 months	0.19 (−0.004, 0.39)	0.056
	Intervention	−0.04(−0.32, 0.25)	0.791
	Sex	−0.24(−0.60, 0.11)	0.177
* Symptoms*	T1-T2: 15 weeks	0.20 (0.03, 0.37)	**0.020**
	T2-T3: 6 months	0.28 (0.09, 0.46)	**0.003**
	Intervention	−0.15(−0.38, 0.08)	0.21
	Sex	0.05 (−0.24, 0.34)	0.733
PROMIS Peer support	T1-T2: 15 weeks	1.09 (−0.60, 2.78)	0.207
	T2-T3: 6 months	−1.19(−2.96, 0.57)	0.187
	Intervention	−0.31(−3.14, 2.51)	0.829
	Sex	−4.77(−8.19,−1.35)	**0.007**

^1^Reference for Time is baseline, representing the time prior to randomization, “Time 1” represents the time at 15-week program completion, and “Time 2” represents the time at 6-month post program completion; ^2^ Reference for intervention group is the control group; ^3^ Reference for sex is male participants. *PROMIS *Patient-Reported Outcomes Measurement Information System, *MEPS *Medical Issues, Exercise, Pain and Social Support, *PedsQL *Pediatric Quality of Life Inventory

However, some sex differences were identified, female participants showed significantly higher level of anxiety (β = 12.54, 95% C.I: 7.02, 18.05; *P* < 0.001) and depression (β = 6.19, 95% C.I: 2.47, 9.91; *P* = 0.001), lower exercise scores on the MEPS (β = −3.59, 95% C.I: −6.47, −0.72; *P* = 0.015) and peer relationships (β = −4.77, 95% C.I: −8.19, −1.35; *P* = 0.007) compared to male participants. By contrast, female participants showed better perceived social ability (β = 3.14, 95% C.I: 0.53, 5.75; *P* = 0.019) compared to male participants.

### Qualitative outcomes—program satisfaction

Nine mentees (8 female, 1 male) and four mentors (3 female, 1 male) agreed to complete the individual interviews or focus group after study completion – reflecting the overall sample, which was predominately female. From these discussions three key categories emerged: (1) Fulfillment and Support Through Shared Experience, (2) Enhancing Program Delivery and (3) Strategies to Boost Engagement. See Table 5 for supportive quotes.

**Table 5:** Supportive quotations of the experiences of mentors and mentees with JIA who participated in the iP2P program

Content Category	Quote
Fulfillment and Support Through Shared Experience	“*We just got along really well. I think she understood what I was going through and she talked about her experiences, we talked about things that like we like to do.*” –14-year old female diagnosed with Polyarthritis
	“*I just wish that this was something that was there when I was a kid, because with something like arthritis, it’s not uncommon for people our age to have it but the first time that I was in a room with people who were like me that had arthritis was when I did the mentoring training. I think it’s so valuable for them to kind of have that representation and to talk to someone who gets it, and that’s not going to kind of like, sugarcoat it in any way.*” – 25-year-old female mentor
Enhancing program delivery	“*I guess maybe with some of the surveys. Not everything on there was completely relevant to me. I don't know if that's something I disliked, it was just something I noticed. Just being like-well, I'm 18, so I'm an adult now. Some of the questions did seem like they were aimed more for a younger audience, I would say.*” – 17-year old female diagnosed with Oligoarthritis
	“*After training, when you get your first mentee, it does kind of feel like you're like just jumping into the deep end, you know, it's a new experience, you have no idea. And obviously mentoring itself is training but I think getting feedback, especially from* *someone who's listening to the calls would have been great in the sense that like it's either like confirmation, “Yeah, you're doing great” or it's like “You can change it up a little bit in this way, and let's try to help a bit”, you know, I think that would be* *really, really helpful*.” – 19-year-old female mentor
	*Someone who, like, gets it and that you can swap stories with a little bit too. And you know, like, share some things that have like happened to you, or like some things that like maybe have happened in mentoring.*” – 25-year old female mentor
	“*I was reading about medications that I had never been on, and so then it was really interesting to kind of go like “Oh, I didn’t even think about that”, or “I didn’t know about that at all”. And so, it might be kind of valuable, in the training, it might feel a bit redundant, but just to go over some things like that. You’re going to be mentoring people who have a very different arthritis experience than you and you won’t know entirely what it’s like for them so that could actually be really valuable.*” – 25-year-old female mentor
Strategies to Boost Engagement	“*I would say like an 8 or a 9. I was excited for the calls and made sure that I was like thinking about any questions that my mentor wanted me to consider through-like between our calls.”* – 17-year old female diagnosed with Oligoarthritis
	“*I had one person that I was mentoring that I was like, “What do you really want to talk about”, and he was like “Dinosaurs?”, and I was like, "Okay, hey, alright”. And so, I did– one time, we did a whole call dedicated to dinosaurs, talking about dinosaurs, but then I kind of like wove it in, because I did research on what dinosaurs had arthritis. Turns out, a couple of dinosaurs actually had arthritis. So I did a little bit of research beforehand and we ended up talking– I somehow bridged, dinosaurs and arthritis together, and so like sometimes that's just how it is, where you're just like, “Okay, you wanna talk about dinosaurs, let’s go for it!*” – 25-year old female mentor

#### Fulfillment and support through shared experience

All interviewed mentees expressed the value of the support, acceptance, and understanding they received through the mentorship program, and reflected positively on their bond with their mentors. The mentoring relationship was facilitated by matching dyads based on similar disease profile and experiences (e.g. pain flare-ups, symptoms, recurrence) as well as non-disease characteristics such as similar interests (e.g., television shows, music, sports, books, and education aspirations). Mentees appreciated not having to justify, or explain their condition, particularly given the stigma of being a young person living with arthritis.

“*I really liked how you could talk to someone, and just kind of, like, share experiences and then just kind of connect to someone who understands what's happening.”* – 16-year old female diagnosed with Oligoarthritis

Many felt it was helpful to have a mentor who was older as it afforded opportunities to receive guidance and advice on disease management, medications, life transitions, and navigating education accommodations in post-secondary settings. Mentees shared they felt more prepared and confident in managing their condition as they transitioned into young adult spaces. Due to these benefits, mentees would recommend the program to their peers with JIA, with two expressing a strong interest in becoming peer mentors themselves in the future.

“*It was really nice because we could talk about university a lot and she could answer my questions on that and just, like, how the transition would be like. So, we ended up having a lot in common with that, and just like general things, we like some of the same books and TV shows, which was super fun. Yeah, I really looked forward to chatting with her.*” –17-year old female diagnosed with Oligoarthritis

Growth and positive experiences with the program were also echoed by mentors. Mentors described how rewarding it was to see their mentees grow over the study period, becoming more positive, understanding, and comfortable with their condition. Mentors emphasized the value for themselves and their mentees in speaking to someone who understands their lived experiences in a way that their families and peers cannot. Most mentors and mentees had never connected with another young person with the same disease prior to the program. Overall, this category is characterized by both mentors and mentees emphasizing the value of peer mentorship in fostering meaningful connections and providing an avenue for effective guidance for adolescents with JIA.

“*The most rewarding part is when you get a kid who is really trying to work through something and sees their disease in a negative light, and that fear, negativity or depression surrounding their disease, and helping them see “Here are the things it’s done for you”, “How you’ve changed as a person because of the experiences that you’ve gone through”, “How it’s given you the determination to do all these things”, “How you now have the perspective to understand different people around you”, and seeing that shift is the most incredible feeling. I found mentoring to be the most important, or valuable thing I’ve done in my life, I feel fulfilled.*” – 24-year-old male mentor

#### Enhancing program delivery

Challenges arose when attempting to schedule and coordinate calls due to differing schedules with their mentor, with some mentees occasionally forgetting to connect with their mentor. Mentees also suggested alternative video platforms to improve program accessibility “*maybe have a FaceTime option because before this program we didn't have Skype”(*16-year old female diagnosed with Oligoarthritis*)*.

While mentors felt the mentor training was excellent, they often spoke about feeling thrown into the ‘*deep end*’ *–* 19-year old female mentor once training was complete, especially the first few calls with their mentees. They felt a lack of direction and assurance on whether they were doing it ‘*right*’ – 19-year old female mentor. They proposed the idea of having check-ins with research staff after the first few calls to debrief and receive feedback to improve future calls. In addition to support from the research team, mentors discussed the value of fostering and maintaining relationships with other peer mentors in the program, which underscored the value of sharing stories and experiences, troubleshooting, gaining new perspectives and learning strategies to better engage with their mentees. Independent of the study, some mentors kept in touch to maintain these unique supportive relationships.

“*But it would have been nice to like, keep a few one-on-one connections, it's less about – I don't think a group chat helps. But I feel like if we were encouraged to swap a few contacts to stay in touch during mentor training, when we're like physically right there or maybe if there was some– some like directory that we could go to that could allow us to keep in touch even.*”* –* 24-year-old male mentor

Mentors requested more JIA disease education during mentor training. They reflected that by learning more about the different JIA disease subtypes, and their respective treatment management plans, they would be better equipped to support mentees with differing disease experiences. This category highlights that although both mentees and mentors found the program beneficial, program delivery could be improved by incorporating ongoing support for mentors and suitable, age-appropriate study materials for mentees.

#### Strategies to boost engagement

All mentees reported high engagement (0–10 scale, with zero being not engaged and 10 being extremely engaged), with responses ranging from 7–10.

Mentors shared strategies they used to promote mentee engagement throughout the program. Some mentors mentioned goal setting in the first few calls to get a better understanding of what mentees are looking to get out of the program. Another mentor mentioned a game they played incorporating ‘get to know you questions’ at the beginning of each session with their mentee. The same mentor also mentioned the need to be creative in designing discussions with mentees using their interests as a guide. Mentors identified engagement differences by genders and developed strategies – through a process of trial and error – that would tailor to the interests to their mentees. For example, one mentor incorporated walking and playing video games to their session to improve engagement with their male mentee. The ability of mentors to critically evaluate their mentorship relationship and actively develop strategies to better engage their mentees is highlighted in this category.

“*Video games are another like thing like one of the boys I mentored was like “Oh, we should play AmongUs and then chat while playing AmongUs”. And it was very strange, and I did it because I was kind of curious how it would go. It didn't go great, I don't know how you could incorporate that, but it's just something that a lot of young boys do a lot of.*” – 24-year-old male mentor

Overall, insights from this qualitative data highlight the utility and perceived benefits of the iPeer2Peer program from the perspective of both mentors and mentees.

## Discussion

JIA is a burdensome condition associated with chronic pain, psychosocial challenges and poor HRQL. Online peer support is a promising avenue to address these difficulties, offering potential improvements in self-management. This study builds on our pilot work, and aimed to assess the clinical effectiveness of the iP2P program among adolescents with JIA through a full-scale RCT. We found no significant improvement in self-management or other clinical effectiveness outcomes. This finding is likely attributable to the study being underpowered, as we did not meet our proposed sample size, likely impacted by the COVID-19 pandemic. Despite this lack of statistical significance, qualitative feedback revealed that both mentors and mentees were satisfied with the program. Mentees valued the opportunity to converse with mentors who empathized with and related to their disease experience, while mentors found the experience fulfilling and noted personal benefits from offering such support.

While our pilot RCT among adolescents with JIA demonstrated improvements in self-management, these findings were not replicated in the present RCT. [21] However, there were major changes between the pilot and the present study (e.g., extended program duration, updated questionnaires) which may explain differing results. The iPeer2Peer program has also been evaluated in several other clinical populations, showing mixed results: improved self-management in adolescents with chronic pain and congenital heart disease respectively, but not in adolescents with sickle cell disease. [22, 23, 36] Online peer mentorship through video conferencing also showed mixed results for improving self-management in other chronic disease populations. [18] Thus, our results align with the broader literature, potential reasons include: recruitment challenges, baseline scores in the normal range from adolescents in the study minimizing room for improvement and limited engagement (i.e., one-third of participants randomized to receive iP2P did not complete a single call) with the program. Recruitment occurred at teaching/academic hospitals with multiple concurrent studies. In order to minimize risk of overwhelming families, some rheumatology clinics prioritized certain research studies over others. This resulted in limited access to the pool of eligible participants, hindering recruitment. Moreover, pandemic related public health restrictions resulted in a complete recruitment pause for several months. Although recruitment eventually resumed (through remote means) recruitment rates did not return to their pre-pandemic levels. High baseline scores may be a reflection of longer disease duration which can result in improved adaptation to JIA. Additionally, recruitment sites were teaching/academic hospitals which may provide more effective disease management, reducing the need for mentorship. This suggests that peer mentorship may be best suited for those who experience greater disease severity or receive care at under-resourced clinical settings. Lower intensity forms of peer support (e.g., online forums, discussion boards) that build a sense of connection and community among adolescents with JIA can be offered more broadly.

While quantitative results did not show improvements in clinical effectiveness outcomes, qualitative findings underscore the value and perceived benefits of the program for both mentors and mentees. Mentees valued the positive and supportive interactions with mentors living with the same condition. Similar results are echoed in other studies exploring peer mentorship among adolescents with JIA and chronic conditions. [18, 21, 22, 36, 37] Connecting through diagnoses allowed mentees to learn more about coping and day-to-day living with their condition and reduced feelings of social isolation. This may be because mentors are closer in age with mentees, facilitating more open and candid conversation than those possible with healthcare providers or family members. Furthermore, the transition to adult care and starting post-secondary education may lead to more in depth conversations between mentees and mentors. Mentors in the program also identified connecting with mentees with a similar condition as a positive experience, building on previous work outlining the positive outcomes associated with being a peer mentor. [37–41] Specifically, mentors felt both fulfilled and supported while assisting adolescents who have undergone similar disease experiences. However, both mentors and mentees mentioned difficulty scheduling mentoring sessions, it is possible that video calling was too burdensome on some adolescents, inclusion of alternative communication avenues such as phone calls and instant messaging (IMs) could alleviate this. Current studies of iP2P have incorporated IMs alongside video calls to address this. [42].

Mentors also provided key suggestions for future improvements, largely around enhanced support structures for them. These suggestions included incorporating more education on different disease experiences and coping methods in the mentor training to support mentoring adolescents with vastly different disease experiences. Our mentor training emphasized the unique role of mentors from other psychosocial providers (e.g., social work, psychology) and encouraged mentors to offer recommendations and coping based on lived experience. Mentors reported feeling apprehensive before and after their initial calls with mentees, however providing them with opportunities to communicate with other mentors in the program or check-in sessions with study staff could ensure that mentors feel supported throughout the duration of the study. While similar initiatives are proposed in other studies, institutional research ethics barriers may vary in guidelines regarding informal communication between mentors. [43, 44] Furthermore, in real-world clinical settings outside the scope of a research, frequent check-ins may hinder adoption and effective implementation of peer mentorship programs. Finally, some mentors found it challenging to connect with male mentees in cross-gender pairings, but proactively addressed this by catering conversations to the mentees’ specific interests. This is well documented in the literature whereby male mentees often find structured verbal mentoring, be it in-person or online, less useful [45]. Males are likely better suited to activity-based mentorship, whereby youth engage with a mentor over a task or game while they talk. [46].

Low engagement, smaller than expected sample size, higher proportion of females and the volume of questionnaire items were limitations for this study. As with many studies, recruiting male participants was challenging. [47] Future iterations of the program should develop approaches that may encourage male mentees to engage in the program and attempt to recruit more male mentors. Although the program was developed with input from adolescents with JIA and pilot tested, this trial still experienced low engagement. This may be attributed to the nature of research, where participants are recruited based on the timing of the study rather than when they are most receptive to guidance/mentorship.

## Conclusion

The iPeer2Peer JIA program is one of the first online peer mentoring programs developed to improve self-management for adolescents with JIA. This clinical trial did not produce significant improvements in self-management or other clinical outcomes. However, qualitative results with mentors and mentees highlight the utility of peer mentoring as both were satisfied with the program and felt the program provided real-word benefits for disease management and well-being. Future research should include more flexibility in mentorship modality (i.e., texting, choice of video platform), identify motivators that enhance adolescent receptiveness to mentorship, include approaches to foster supportive environment for mentors, and could seek to ascertain whether a tailored approach by gender or disease severity would enhance engagement with peer-mentorship programs.

## Data Availability

The raw data will not be made available in order to protect against the possible identification of any patients who took part in the study. As per institution REB, any release of data requires contracts outlining the recipient of data and purposed use of data.

## Abbreviations

JIA: Juvenile Idiopathic Arthritis
HRQL: Health related quality of life
RCT: Randomized control trial
SickKids: The Hospital for Sick Children
ILAR: International League of Associations for Rheumatology
SD: Standard deviation
IMs: Instant messaging
